# Intention to Use a Mental Health App for Menopause: Health Belief Model Approach

**DOI:** 10.2196/60434

**Published:** 2024-10-16

**Authors:** Nayra A Martin-Key, Erin L Funnell, Jiri Benacek, Benedetta Spadaro, Sabine Bahn

**Affiliations:** 1 Cambridge Centre for Neuropsychiatric Research Department of Chemical Engineering and Biotechnology University of Cambridge Cambridge United Kingdom

**Keywords:** menopause, menopause transition, mental health, perimenopause, women’s health, psychological framework, symptom tracking, app usage, app, Health Belief Model

## Abstract

**Background:**

Menopause presents a period of heightened vulnerability for mental health issues. Despite this, mental health screening is not consistently integrated into menopausal health care, and access to psychological interventions is limited. Digital technologies, such as web and smartphone apps, may offer a way to facilitate and improve mental health care provision throughout menopause. However, little is known about potential users’ intention to use such technologies during this critical phase of life.

**Objective:**

To examine the factors that impact the intention of potential users to use a mental health app during menopause, we used the Health Belief Model (HBM), a psychological framework widely used to understand and predict individuals’ health-related behaviors.

**Methods:**

An online survey was generated. Convenience sampling was used, with participants recruited via social media and email, through relevant foundations and support groups, and by word of mouth. Structural equation modeling with maximum likelihood estimation was conducted to explore whether the factor structure of the HBM is a good fit for predicting the intention to use a mental health app for menopause. A Cronbach α value of .05 was used for determining statistical significance.

**Results:**

A total of 1154 participants commenced the survey, of which 82.49% (n=952) completed at least 97% of the survey. Of these, 86.76% (n=826) expressed that their menopausal symptoms had negatively affected their mental health, and went on to answer questions regarding their experiences and interest in using a web or smartphone app for mental health symptoms related to menopause. Data from this subgroup (N=826) were analyzed. In total, 74.09% (n=612) of respondents sought online help for mental health symptoms related to menopause. The most common topics searched for were symptom characteristics (n=435, 52.66%) and treatment or therapy options (n=210, 25.42%). Psychoeducation (n=514, 62.23%) was the most desired mental health app feature, followed by symptom tracking (n=499, 60.41%) and self-help tips (n=469, 56.78%). In terms of the intention to use a mental health app, the Satorra-Bentler–scaled fit statistics indicated a good fit for the model (χ2278=790.44, *P*<.001; comparative fit index=0.933, root mean square error of approximation=0.047, standardized root mean square residual=0.056), with cues to action emerging as the most significant predictor of intention (β=.48, *P*<.001). This was followed by perceived barriers (β=–.25, *P*<.001), perceived susceptibility (β=.15, *P*<.001), and perceived benefits (β=.13, *P*<.001). Perceived severity (β=.01, *P*=.869) and self-efficacy (β=.03, *P*=.286) were not significantly associated with behavioral intention.

**Conclusions:**

This study reveals important factors that influence the intention to use a mental health app during menopause. It emphasizes the need to address barriers to app usage, while highlighting the impact of credible endorsements and psychoeducation. Furthermore, the study underscores the significance of improving accessibility for users with lower digital literacy or limited resources.

## Introduction

Menopause represents a significant milestone in a woman’s life (throughout the study, we refer to a woman as anyone assigned female at birth). It tends to occur naturally between the ages of 44 and 55 years [1], marking the cessation of menstruation due to the decline in ovarian follicular function [2]. Perimenopause, also known as the menopause transition, refers to the phase leading up to menopause and is characterized by a gradual decrease in ovarian function, resulting in less frequent menstrual cycles. This transitional period is estimated to last a median of 4 years [3], signifying an important and transformative stage in a woman’s reproductive journey.

Every woman’s journey through menopause is unique, but the associated symptoms and the transitional phase can be incredibly challenging. Indeed, the menopause and perimenopause phases are typically associated with physical symptoms (eg, hot flushes, bone and joint pain, loss of libido) that can have a significant impact on an individual’s quality of life [4]. In addition, menopause, and particularly the menopause transition, can increase vulnerability to mental health issues [5], particularly depression and anxiety [6-8], as well as suicidal ideation [9]. Critically, despite several professional bodies recommending psychological interventions as a primary treatment option for menopause-related mental health concerns [10-12], access to these treatments is often limited [13], even in high-income countries, such as the United Kingdom. There is also evidence to suggest that mental health screening is not consistently integrated into menopausal health care in the United Kingdom [14], indicating a missed opportunity to identify and address potential mental health concerns that may arise during this challenging phase of life.

In this regard, digital technologies, such as web and smartphone apps, may offer a cost-effective and highly scalable way to facilitate and improve mental health care provision in the United Kingdom throughout menopause and the menopause transition. There is evidence to suggest that digital platforms have the potential to enhance the reach, quality, and effectiveness of mental health care services [15], with the use of digital apps for screening and monitoring of mental health symptoms showing promising results across various mental health conditions [16]. Additionally, evidence suggests that individuals are more inclined to disclose severe symptoms on technology platforms than to a health care professional (HCP) [17], and patients appear to value the autonomy and empowerment gained through the use of digital platforms [18]. Recent evidence also suggests that virtual interventions have the potential to improve both physical and psychosocial outcomes of menopausal women [19], but little is known about UK-based perimenopausal and menopausal women’s *intention* to use digital technologies for mental health concerns that may arise during this critical phase of life.

To this end, we set out to explore (1) experiences with and preferences toward mental health apps for mental health concerns related to menopause in the United Kingdom and (2) factors that may influence potential users’ intention to use a mental health app throughout menopause and the menopause transition. To achieve the latter, we used the Health Belief Model (HBM [20]), a psychological framework that seeks to explain and predict individuals’ health-related behaviors. The HBM consists of several key components, namely perceived susceptibility (an individual’s perception of their vulnerability or likelihood of experiencing a particular health condition), perceived severity (an individuals’ belief about the seriousness and potential consequences of a health condition), perceived benefits (an individuals’ beliefs in the effectiveness and positive outcomes of adopting a health-related behavior), perceived barriers (an individual’s assessment of obstacles, costs, or negative aspects associated with adopting a health-related behavior), cues to action (triggers that prompt an individual to take action toward a particular health-related behavior), and self-efficacy (an individual’s belief in their ability to successfully perform a specific health-related behavior).

he HBM has been widely used to understand and promote health behaviors in various contexts, including disease prevention [21], health promotion [22], adherence to medical treatments [23], and health behaviors during menopause [24,25]. The key objective of this study was to understand the health belief constructs that may influence United Kingdom women’s intention to use a mental health app throughout menopause and the menopause transition. The secondary objective of this study was to explore online help-seeking behaviors and preferences in app features in women with experience of menopause-related mental health concerns in the United Kingdom (UK). The findings from this study have important implications for the development of effective ways to provide digital mental health care solutions throughout this complex time.

## Methods

### Overview

This study used data from a UK-wide anonymous online survey study conducted by the Cambridge Centre for Neuropsychiatric Research between January and March 2023 [14]. The key objectives of the study were to (1) understand the current state of care provision offered via health care services in the United Kingdom throughout menopause and the menopause transition and (2) explore the use of and interest in digital technologies for mental health throughout menopause and the menopause transition. To this end, an anonymous online survey was created using Qualtrics XM. The survey could be completed in 15-20 minutes and comprised 5 sections: (1) sociodemographic information, (2) health care provision throughout menopause, (3) mental health symptoms and care provision throughout menopause, (4) menopause-specific quality-of-life symptoms, and (5) experiences and interest in using a web or smartphone app for mental health symptoms related to menopause.

The latter section also included 26 items pertaining to HBM constructs (see Table 1 for a description of the constructs and list of items in the study). Items were developed based on previous research [26,27] and in consultation with a practicing psychiatrist (author SB) and a patient and public involvement (PPI) panel of members with lived experience of mental health concerns. Items were rated on a 6-point Likert-scale, from 1 (strongly disagree) to 6 (strongly agree). For the purpose of this study, only data from sections 1 and 5 were included. The survey was adaptive in nature, meaning that only relevant questions were asked based on responses to previous questions.

**Table 1:** HBM^a^ constructs (ie, perceived benefits, perceived severity, perceived susceptibility, perceived barriers, cues to action, self-efficacy, behavioral intention), their descriptions, and respective 26 items used in the study.

HBM construct	Description	Items
Perceived benefits	The perceived benefits associated with using a mental health app for mental health symptoms related to menopause	A better understanding of my mental health symptoms would prevent problems with friends and family. A burden would be lifted off me if I better understood my mental health symptoms. A better understanding of my mental health symptoms would encourage me to seek professional help.
Perceived severity	The perceived severity of mental health symptoms related to menopause	My mental health symptoms are serious. My mental health symptoms have negative consequences on my life. My mental health symptoms cause difficulties for those who are close to me.
Perceived susceptibility	The perceived likelihood or vulnerability of experiencing mental health symptoms related to menopause (eg, low mood)	My family history puts me at risk for mental health disorders. My lifestyle puts me at risk for mental health disorders. The amount of stress in my life puts me at risk for mental health disorders. Menopause puts me at risk for mental health disorders.
Perceived barriers	The perceived obstacles associated with using a mental health app for mental health symptoms related to menopause	I am not comfortable getting my symptoms assessed by a mental health app. I think knowing the results of my mental health assessment would be too distressing. I think that a mental health app would not be able to understand my symptoms. I worry about the mental health app keeping my data confidential. I worry about the potential costs related to the app and seeking mental health support.
Cues to action	A prompt that would encourage use of a mental health app for mental health symptoms related to menopause	I would use the mental health app if it were developed and validated by psychiatrists. I would use the mental health app if it were developed by a reputable university. I would use the mental health app if an HCP^b^ (eg, my GP^c^) or the NHS^d^ recommended it. I would use the mental health app if a friend or family member recommended it. I would use the mental health app if it were advertised on social media (Facebook, Instagram, Twitter, YouTube, etc).
Self-efficacy	A belief in possessing the necessary resources and skills to use a mental health app for mental health symptoms related to menopause	I know how to download an app or access a website. I have the necessary resources to use a mental health website or app (eg, computer, smartphone, internet connection). I can get help from others (eg, family, or others) if I am having difficulties using an app or website.
Behavioral intention	The intention to use a mental health app for mental health symptoms related to menopause	I would be willing to try the mental health app. I plan to try the mental health app once it becomes available. I want to use the mental health app in the future.

^a^HBM: Health Belief Model.

^b^HCP: health care professional.

^c^GP: general practitioner.

^d^NHS: National Health Service.

### Participants

Convenience sampling was used, with participants recruited between January and March 2023 via email, paid Facebook and Instagram advertisements, organic posts on the Cambridge Centre for Neuropsychiatric Research Facebook and X (formerly known as Twitter) pages, and Reddit. Recruitment messages were also disseminated by word of mouth and through relevant foundations and support groups. Inclusion criteria for the study were as follows: (1) age≥18 years, (2) UK residence, and (3) must be *currently* experiencing symptoms of menopause or the menopause transition (eg, hot flushes, mood changes, night sweats, irregular or absent periods, decreased sex drive). Participants were also required to *not* be currently pregnant or breastfeeding and *not* have been diagnosed with a mental health condition in order to take part in the study. Participants of any gender (ie, female, male, nonbinary, other, those preferring not to answer) could take part in the study, provided that they met the inclusion criteria (note that we used the term “women” to refer to all participants in the study).

Menopause status was based on the definitions put forth by the Study of Women’s Health Across the Nation (SWAN [28]). Those in the menopause transition included individuals who self-reported being in the early or late perimenopause stages (ie, those experiencing significant changes in their menstrual cycles not due to pregnancy, breastfeeding, stress, or a medical condition or who had not had menstrual bleeding for 3-11 months not due to pregnancy, breastfeeding, stress, or a medical condition). Those in menopause had undergone 12 months without menstrual bleeding not due to pregnancy, breastfeeding, or stress, as well as those who had undergone medically induced menopause.

Participants were invited to enter their email for the chance to win 1 of 3 GB £50 (US $67) Highstreet vouchers. Participants were able to withdraw from the study at any point.

### Data Analysis

Descriptive statistics were conducted in IBM SPSS version 28.0.1.1. Figures were created using Microsoft Excel version 2206 and Microsoft PowerPoint version 2206 (Microsoft Office 365). HBM data were analyzed in Stata version 17.0 (StataCorp) [29]. Mean scores per HMB construct were obtained by summing the corresponding HBM constructs and dividing the sum by the number of items. Structural equation modeling (SEM) with maximum likelihood estimation was conducted to explore whether the factor structure of the HBM is a good fit for predicting the intention to use a digital mental health app for mental health–related symptoms that can arise as a result of menopause or the menopause transition. All exogenous latent variables were assumed to be correlated. The Satorra-Bentler–scaled *χ*^2^ test for model goodness-of-fit evaluation was reported as data were nonnormally distributed (assessed using the Doornik-Hansen test). Although *χ*^2^ is commonly reported to evaluate fit, and a good model should present with a *P* value above the 0.05 threshold [30], it is sensitive to sample size. As such, it is not necessarily a reliable basis for the acceptance or rejection of a model [31-33]. For this reason, we also evaluated the model fit using the comparative fit index (CFI), the root mean square error of approximation (RMSEA), and the standardized root mean square residual (SRMR), as is the recommendation [34]. A good model fit for the purposes of the study satisfied a CFI value of 0.90, an RMSEA below 0.60, and an SRMS of less than 0.08 [35]. A Cronbach α value of 0.05 was used for determining statistical significance.

### Ethical Considerations

The study was approved by the University of Cambridge Human Psychology Research Ethics Committee (approval number PRE.2022.110). All participants provided informed consent electronically to participate in the study.

## Results

### Sociodemographic Characteristics

Participants’ sociodemographic information across the entire sample can be found in Table 2. A total of 1154 participants commenced the survey, of which 82.49% (n=952) completed at least 97% of the survey (this completion rate ensured no missing data for the analysis of interest). Of these, 86.76% (n=826) expressed that their menopausal symptoms had negatively affected their mental health, and went on to answer questions in section 5 (ie, experiences and interest in using a web or smartphone app for mental health symptoms related to menopause). Data from this subgroup (N=826) were analyzed. The average age was 50.28 (SD 5.08) years, with the majority of respondents self-identifying as female (n=811, 98.18%), being White (n=803, 97.22%), having at least an undergraduate degree (n=501, 60.65%), and being married or in a civil partnership (n=518, 62.71%). Regarding accommodation characteristics, living with a partner and children (n=375, 45.39%) and living with a partner (n=281, 34.02%) were the most common arrangements. In addition, 85.47% (n=706) were employed, and 61.14% (n=505) had a household income of at least GB £35,001 (US $46,787) before tax.

**Table 2:** Sociodemographic characteristics of the sample surveyed in the study (N=826).

Characteristics		Value
Age (years), mean (SD)		50.28 (5.08)
**Gender identity, n (%)**		
	Female	811 (98.18)
	Male	1 (0.12)
	Nonbinary	5 (0.61)
	Other	7 (0.85)
	Prefer not to answer	2 (0.24)
**Ethnicity, n (%)**		
	White	803 (97.22)
	Asian/Asian British	4 (0.48)
	Black/African/Caribbean/Black British	1 (0.12)
	Mixed/multiple ethnic groups	16 (1.94)
	Arab	2 (0.24)
**Education, n (%)**		
	Below GCSE^a^/equivalent	17 (2.06)
	GCSE/equivalent	122 (14.77)
	A level^b^/IB^c^/advanced higher	169 (20.46)
	Undergraduate degree	244 (29.54)
	Postgraduate degree	257 (31.11)
	Other	14 (1.69)
	Prefer not to answer	3 (0.36)
**Relationship status, n (%)**		
	Single	88 (10.65)
	Married/civil partnership	518 (62.71)
	Cohabiting	134 (16.22)
	Separated	15 (1.82)
	Divorced	51 (6.17)
	Other	19 (2.30)
	Prefer not to answer	1 (0.12)
**Living arrangement, n (%)**		
	Living alone	87 (10.53)
	Living in shared accommodation with previously unknown individual(s)	1 (0.12)
	Living with relative(s), including single parent	82 (9.93)
	Living with a partner	281 (34.02)
	Living with a partner and children	375 (45.40)
**Employment^d^, n (%)**		
	Full-time employment	394 (47.70)
	Part-time employment	220 (26.63)
	Self-employment	92 (11.14)
	Parental leave/care for a family member	30 (3.63)
	Student	15 (1.82)
	Voluntary work	15 (1.82)
	Retired	35 (4.24)
	Unemployed	57 (6.90)
	Prefer not to answer	13 (1.57)
**Household income (GB £)^e^, n (%)**		
	<15,000 (US $20,051)	61 (7.38)
	15,001-25,000 (US $20,052-$33,418)	60 (7.26)
	25,001-35,000 (US $33,420-$46,786)	100 (12.11)
	35,001-45,000 (US $46,787-$60,153)	85 (10.29)
	45,001-55,000 (US $60,154-$73,520)	94 (11.38)
	55,001-65,000 (US $73,522-$86,888)	75 (9.08)
	65,001-75,000 (US $86,889-$100,255)	79 (9.56)
	75,001-85,000 (US $100,256-$113,622)	54 (6.54)
	>85,001 (US $113,624)	118 (14.29)
	Prefer not to answer	100 (12.11)

^a^A level: advanced-level qualification.

^b^GCSE: General Certificate of Secondary Education.

^c^IB: International Baccalaureate.

^d^Percentages may add to more than 100 as participants could select multiple options.

^e^An exchange rate of 1 GB £=US $1.34 was applied.

### Experiences and Interest in Using Digital Technology for Mental Health Symptoms Related to Menopause

**Figure 1:**
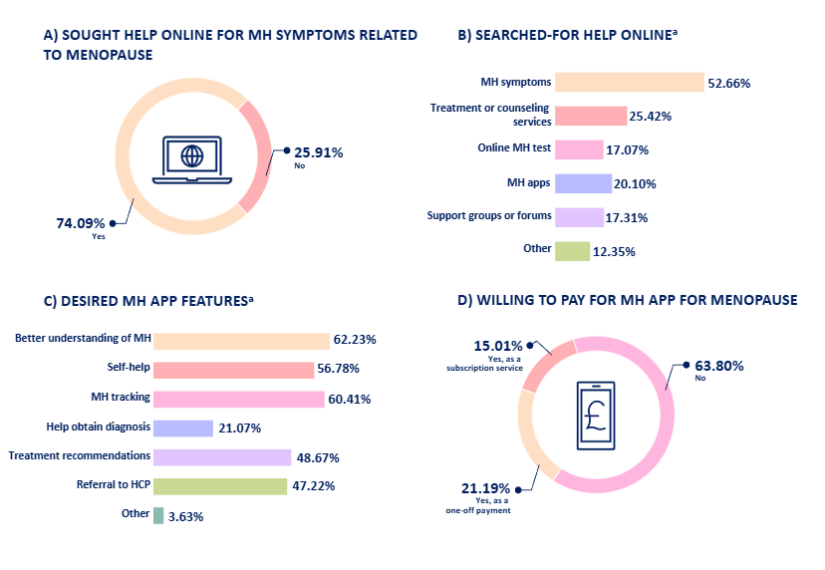
Respondents’ experiences and interest in using digital technology for mental health symptoms related to menopause (N=826), including (A) sought help online for MH symptoms related to menopause, (B) searched for help online, (C) desired MH app features, and (D) willing to pay for MH app for menopause. aPercentages may add up to more than 100 as respondents could select multiple options. HCP: health care professional; MH: mental health.

A summary of respondents’ experiences and interest in using digital technology for mental health symptoms related to the menopause can be found in Figure 1. In total, 74.09% (n=612) of respondents had sought help online regarding mental health symptoms related to menopause (Figure 1A), with the help most commonly searched for including looking for information about symptom characteristics (n=435, 52.66%) and treatment or therapy options (n=210, 25.42%), as shown in Figure 1B. When respondents were asked to select features for their ideal mental health app, the most sought for feature was psychoeducation (ie, gaining a better understanding of one’s mental health state; n=514, 62.23%), as shown in Figure 1C. This was closely followed by the capability to track symptoms over time (n=499, 60.41%) and self-help tips (n=469, 56.78%), as shown in Figure 1C. When asked about willingness to pay for a mental health assessment app, 63.80% (n=527) of respondents said they would *not* be willing to pay, while 21.19% (n=175) said they would pay as a one-off service and 15.01% (n=124) as a subscription-based service (Figure 1D).

### Structural Equation Modeling

Descriptive statistics for the HBM constructs, including internal consistency scores (overall Cronbach α=.74) can be found in Table 3, with a correlation matrix of the constructs presented in Table 4. The mean behavioral intention score was 4.51 (SD 1.14), indicating an interest in using an app for mental health symptoms related to menopause, and behavioral intention was seen to be significantly correlated to all HBM constructs (all r=0.20-0.66, all *P*<.01). Perceived barriers were negatively correlated with behavioral intention (r=–0.40, *P*<.01).

**Table 3:** Mean scores and internal consistency scores (ie, Cronbach α) of HBM^a^ constructs in the study (N=826).

HBM construct	Mean (SD)	Cronbach α^b^
Perceived benefits	4.12 (1.13)	0.80
Perceived severity	3.90 (1.30)	0.81
Perceived susceptibility	3.48 (1.08)	0.63
Perceived barriers	2.83 (1.00)	0.66
Cues to action	4.08 (0.86)	0.75
Self-efficacy	5.53 (0.63)	0.66
Behavioral intention	4.51 (1.14)	0.94

^a^HBM: Health Belief Model.

^b^High reliability, α≥.80; moderate reliability, α=.50-.80; low reliability, α<.50.

**Table 4:** Correlation matrix of HBM^a^ constructs in the study (N=826).

HBM construct	Perceived benefits	Perceived severity	Perceived susceptibility	Perceived barriers	Cues to action	Self-efficacy	Behavioral intention
Perceived benefits	—^b^	0.20^c^	0.11^c^	–0.11^c^	0.35^c^	0.05	0.38^c^
Perceived severity	—	—	0.45^c^	–0.00	0.16^c^	0.01	0.23^c^
Perceived susceptibility	—	—	—	0.04	0.17^c^	0.00	0.20^c^
Perceived barriers	—	—	—	—	–0.36^c^	–0.18^c^	–0.40^c^
Cues to action	—	—	—	—	—	–0.23^c^	0.66^c^
Self-efficacy	—	—	—	—	—	—	0.23^c^

^a^HBM: Health Belief Model.

^b^Not applicable.

^c^*P*<.01.

**Figure 2:**
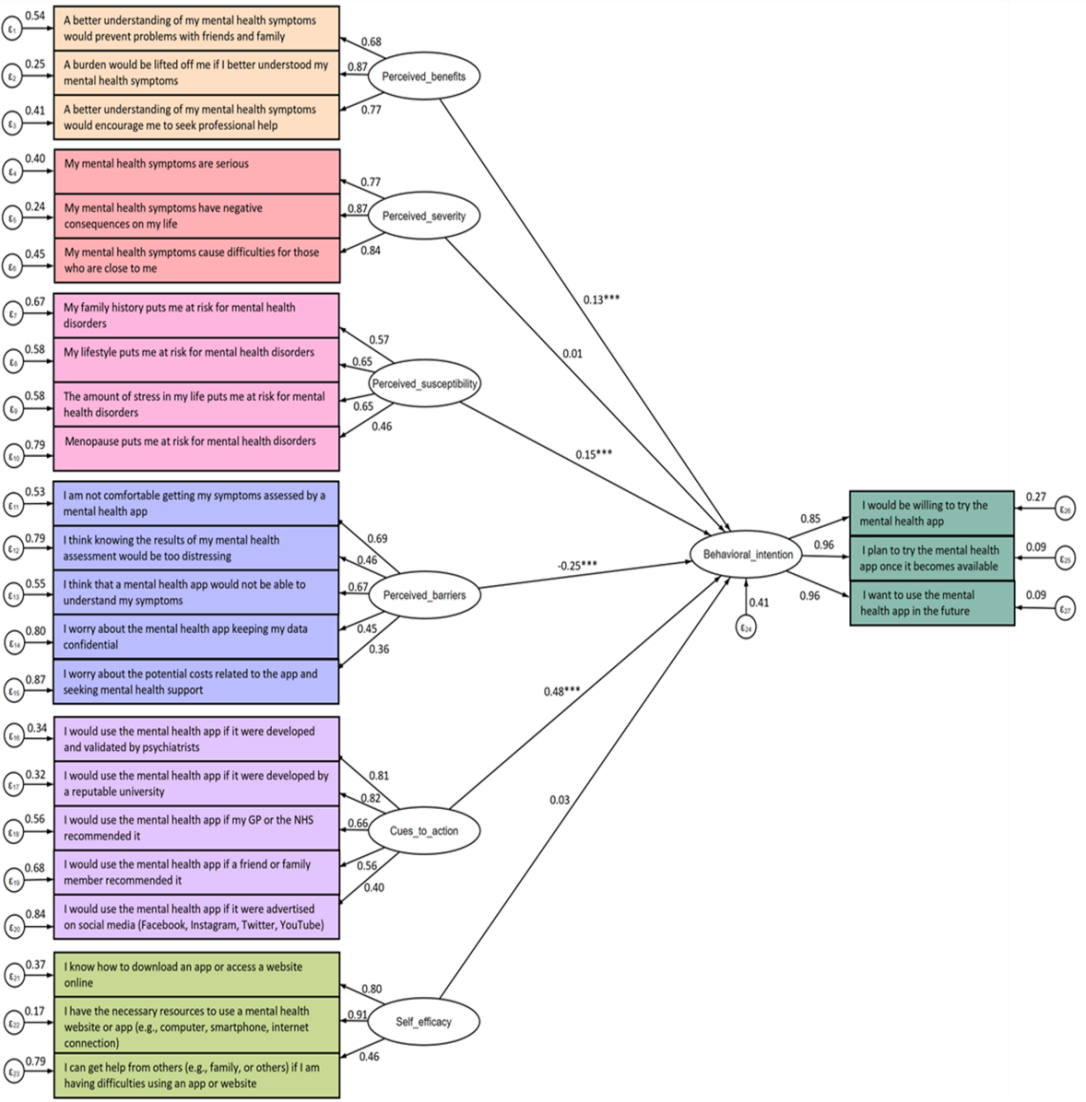
SEM schema of HBM constructs predicting the intention to use an app for mental health symptoms related to menopause. ***P<.001; GP: general practitioner; HBM: Health Belief Model; NHS, National Health Service; SEM, structural equation modeling.

Results of the Satorra-Bentler–scaled fit statistics indicated a good model fit (*χ*^2^_278_=790.44, *P*<.001; CFI=0.933, RMSEA=0.047, SRMR=0.056). The model schema and results are presented in Figure 2 (covariates have been removed for readability purposes; see Figure S1 in Multimedia Appendix 1 for the model schema including covariates). All factor loadings between our observed and latent variables were significant and ≥0.36.

Our analyses revealed that the constructs of perceived severity, perceived susceptibility, perceived benefits, perceived barriers, cues to action, and self-efficacy explained 58.59% of the variance in behavioral intention. The most important predictor of intention was cues to action (β=.48, *P*<.001; eg, the app being developed by psychiatrists or a reputable academic institution, the app being recommended by an HCP or the National Health Service [NHS]). This was followed by perceived barriers (β=–.25, *P*<.001; eg, not feeling comfortable having symptoms assessed by a mental health app, the app not being able to understand mental health symptoms related to menopause), which was negatively associated with behavioral intention. Perceived susceptibility (β=.15, *P*<.001; eg, lifestyle factors and life stressors increasing one’s vulnerability to mental health symptoms related to menopause) and perceived benefits (β=.13, *P*<.001; eg, an improved understanding of menopause-related mental health symptoms having the potential to alleviate the burden of mental health symptoms and encourage formal help-seeking behavior) were also significant predictors of behavioral intention. However, perceived severity (β=.01, *P*=.869; eg, mental health symptoms related to menopause having negative consequences on one’s life) and self-efficacy (β=.03, *P*=.286; eg, having the necessary resources to use a mental health app) were not significantly associated with behavioral intention.

## Discussion

### Principal Findings

The key objective of this study was to explore women’s intention to use a mental health app throughout menopause and the menopause transition. The secondary objective of this study was to explore previous online help-seeking behaviors and preferences in features for apps designed for women with experience of menopause-related mental health concerns. Overall, the vast majority of women had turned to online resources for assistance with mental health concerns associated with menopause. The type of help most frequently searched for online included gaining information about symptom characteristics and exploring treatment or therapy options. When asked about their preferred features in a mental health app, psychoeducation, which involves gaining a better understanding of one’s mental health condition, was the most frequently selected feature. This was closely followed by the ability to track symptoms over time and access self-help tips. In regard to the intention to use an app based on the HBM, we found that cues to action was the strongest predictor of women’s intention to adopt the app. This was followed by perceived barriers, perceived susceptibility, and perceived benefits. On the other hand, perceived severity and self-efficacy were not significantly associated with intention to use the app.

Regarding cues to action, the importance of a mental health app being developed by psychiatrists or a reputable academic institution were recognized as key factors driving cues to action and, in turn, the intention to use the app, supporting previous research [36]. Additionally, receiving active recommendations for an app from HCPs was identified as an excellent strategy to encourage adoption, aligning with previous studies [37]. These findings emphasize the significance of establishing strong collaborations between app developers, reputable organizations (eg, the NHS and academic institutions), and HCPs. Actively involving these partners in the development, promotion, and endorsement of a mental health app is likely to significantly enhance positive attitudes and intentions toward its adoption. Moreover, leveraging the authority and influence of HCPs and health care systems can help address concerns or skepticism regarding the app’s effectiveness, reliability, and suitability for managing mental health symptoms related to menopause.

Perceived barriers, such as discomfort with an app assessing one’s mental health symptoms and doubts about the app’s ability to understand the complexity of mental health issues, were identified as significant barriers for app usage. Interestingly, evidence indicates that individuals tend to feel more at ease disclosing sensitive health information digitally than to an HCP [17]. However, app developers must be mindful of fostering trust by providing clear and transparent app descriptions and privacy policies to users [38,39], as not doing so can inadvertently create a sense of mistrust. Research also suggests that users feel more comfortable with certain app features, such as appointment reminders, compared to passive data-tracking features, such as GPS and call/text log monitoring [40]. Therefore, app developers need to investigate perceptions of trust of specific app features in their population of interest. Regarding concerns about an app’s ability to understand mental health symptoms, developers should thoroughly assess its effectiveness and feasibility in the intended population [41]. This approach ensures the creation of a high-quality evidence-based assessment and fosters trust among users. Consequently, HCPs can rely on this evidence to confidently suggest or refer to clinically safe and effective technologies.

Regarding perceived susceptibility, women who believed they were more susceptible to mental health symptoms were more likely to express an intention to use a mental health app. In particular, women who viewed their lifestyle and life stressors as key drivers of poor mental health were more likely to state an interest in using an app. Indeed, there is a well-established correlation between modifiable lifestyle factors and poor mental health during menopause. For instance, having a high BMI and leading a sedentary lifestyle are both linked to increased odds of experiencing mental health issues [42,43]. Additionally, a recent systematic review revealed that stressful life events occurring during menopause, including illness, marital discord, and situations where children leave home or face difficulties in pursuing higher education or finding employment, are linked with increased rates of depression and anxiety during this transitional phase [44]. Considering these findings, app developers may find it beneficial to adopt a holistic approach to mental health care by providing women with tips on maintaining a healthy lifestyle and reducing stress during this challenging phase of life, as the inclusion of such features is likely to be attractive to those most willing to use a mental health app.

When considering the perceived benefits of using a mental health app, an improved understanding of menopause-related mental health symptoms was found to have the potential to alleviate the burden of mental health symptoms, as well as encourage formal help-seeking behavior. In this regard, increased awareness and discussions about menopause and its associated mental health implications in public health campaigns and the media can facilitate women’s understanding of their vulnerability to these symptoms and, in turn, motivate them to adopt a mental health app as a proactive measure for self-care and mental health symptom management. Notably, research has highlighted that a better understanding and awareness of menopause and its transition allow women to feel more empowered to make better health care decisions during this phase of life [45]. Similarly, providing individuals with information about mental health symptoms and conditions via the means of psychoeducation, for instance, can increase symptom knowledge and has been demonstrated to boost intention to seek help, as well as improve patient engagement and adherence to HCPs’ recommendations [39,46,47]. It is crucial, therefore, for app developers to explore potential collaborations with public health bodies or the media who are delivering menopause education, as well as identify any opportunities to empower individuals with high-quality evidence-based psychoeducation resources that support women’s mental health during menopause and the menopause transition through increased knowledge and signposting to services.

In this study, both perceived severity and self-efficacy were *not* significant drivers of app usage. Regarding the former, it is often assumed that individuals who perceive their symptoms as more severe would be more motivated to use a mental health app as a means of managing and addressing their symptoms. However, contrary to this expectation, the study findings did not support a significant relationship between perceived severity and the intention to use a mental health app. Notably, previous studies have reported similar findings, indicating that perceived severity does not significantly predict health-related behaviors in various contexts. For instance, research has shown that perceived severity is not a significant factor in determining behaviors such as facemask use [48], vaccine uptake [49], and adoption of contact-tracing apps [26]. In addition, a qualitative study investigating methods of optimizing smartphone apps for cardiovascular disease did not find perceived severity to be a key driver of app usage [50].

In terms of self-efficacy, the majority of women in this study expressed confidence in their ability to use a mental health app and had the necessary resources for its use. This confidence can be attributed to the widespread prevalence of smartphones in the United Kingdom, and it is likely that the study sample consisted of individuals with a high level of digital literacy. Although perceived self-efficacy had no influence on the intention to use a mental health app, app developers should focus on strategies that promote sustained app engagement. Improving ease of use and providing in-app guidance have proven effective in increasing app usage [51]. Furthermore, to ensure inclusivity and reach individuals who may benefit from an app but lack the necessary resources, developers should consider incorporating features such as offline functionality or the option to complete a mental health assessment via text messaging. These measures have the potential to enhance accessibility, particularly for hard-to-reach women.

### Limitations

The participants in this study exhibited characteristics that differed from the general UK population in several ways. They had higher levels of education, a higher household income, and a higher proportion of White individuals. As a result, it is important to acknowledge that the views expressed in this study may not provide a comprehensive representation of the broader UK population. Specifically, the perspectives of ethnic minorities and disadvantaged populations in the United Kingdom, who may face additional barriers or have different attitudes toward using digital solutions for menopause-related concerns, may be underrepresented. Furthermore, it is worth noting that the primary method of participant recruitment for this study was through social media channels. This means that the respondents in this study are more likely to possess high levels of digital literacy skills and may have a greater inclination to use digital tools for health care purposes. Considering these factors is crucial when interpreting the findings of this study and extending them to the wider UK population.

Of note, although the HBM constructs included in the study were codesigned with a psychiatrist and reviewed by individuals with lived experience of mental health concerns, there is a possibility that some items may not fully capture the experiences of individuals with mental health symptoms related to menopause or that certain important aspects may have been missed. Additionally, since the HBM constructs were developed specifically for this study and have not undergone prior validation, there is a potential for measurement inaccuracies and biases in the findings. As a result, the study’s conclusions should be interpreted with this limitation in mind.

In addition, it is worth noting that the data were nonnormally distributed, with a high proportion of respondents selecting the lowest and highest response options. Although our analyses accounted for this, this nonnormality may still have implications for the interpretability of our model. For instance, the presence of ceiling and floor effects may have reduced the variability in our data, potentially obscuring more subtle relationships between variables. This could limit the ability of the model to fully capture the complexity of the constructs under investigation.

### Conclusion

This study sheds light on behavioral drivers influencing the intention to use a mental health app during menopause and the menopause transition. Notably, credible endorsements from reputable sources, addressing perceived barriers, such as concerns about the efficacy of a mental health app, and enhancing mental health literacy through psychoeducation, emerged as significant factors in encouraging app usage. App developers should consider these insights during development and promotion to create apps that can positively impact mental health during this challenging life phase. Additionally, considering features that enhance accessibility for users with lower digital literacy or limited resources will ensure inclusivity and reach a broader audience. By integrating these strategies, app developers can offer valuable support and care to those facing mental health challenges related to menopause and contribute to their overall well-being.

## Appendix

Multimedia Appendix 1 Model schema of HBM constructs predicting intention to use an app for mental health (including all covariates). HBM: Health Belief Model.

## Abbreviations

CFI: comparative fit index
HBM: Health Belief Model
HCP: health care professional
NHS: National Health Service
RMSEA: root mean square error of approximation
SEM: structural equation modeling
SRMR: standardized root mean square residual

## Data Availability

The data sets used and analyzed during this study are available from the corresponding author upon reasonable request.

## References

[1] Hardy C, Hunter M, Griffiths A (2018). Menopause and work: an overview of UK guidance. Occup Med (Lond).

[2] (2008). World Health Report 2008: women, ageing and health: a framework for action. World Health Organization.

[3] Delamater L, Santoro N (2018). Management of the perimenopause. Clin Obstet Gynecol.

[4] Dave FG, Adedipe T, Disu S, Laiyemo R (2019). Unscheduled bleeding with hormone replacement therapy. Obstetric Gynaecol.

[5] Soares CN (2019). Depression and menopause: an update on current knowledge and clinical management for this critical window. Med Clin North Am.

[6] Freeman E (2010). Associations of depression with the transition to menopause. Menopause.

[7] Bromberger JT, Kravitz HM, Chang Y, Cyranowski JM, Brown C, Matthews KA (2011). Major depression during and after the menopausal transition: Study of Women's Health Across the Nation (SWAN). Psychol Med.

[8] Hart J (2019). Menopause: shifting hormones linked to anxiety and depression symptoms. Altern Complement Ther.

[9] Usall J, Pinto-Meza A, Fernández A, de Graaf R, Demyttenaere K, Alonso J, de Girolamo G, Lepine JP, Kovess V, Haro JM (2009). Suicide ideation across reproductive life cycle of women. Results from a European epidemiological study. J Affect Disord.

[10] Stute P, Spyropoulou A, Karageorgiou V, Cano A, Bitzer J, Ceausu I, Chedraui P, Durmusoglu F, Erkkola R, Goulis DG, Lindén Hirschberg A, Kiesel L, Lopes P, Pines A, Rees M, van Trotsenburg M, Zervas I, Lambrinoudaki I (2020). Management of depressive symptoms in peri- and postmenopausal women: EMAS position statement. Maturitas.

[11] Maki P, Kornstein S, Joffe H (2018). Guidelines for the evaluation and treatment of perimenopausal depression: summary and recommendations. Menopause.

[12] (2015). Menopause: diagnosis and management. NICE diagnostic guidance NG23. National Institute for Health and Care Excellence.

[13] Fenlon D, Morgan A, Khambaita P, Mistry P, Dunn J, Ah-See M, Pennery E, Hunter MS (2017). Management of hot flushes in UK breast cancer patients: clinician and patient perspectives. J Psychosom Obstet Gynecol.

[14] Martin-Key NA, Funnell EL, Spadaro B, Bahn S (2023). Perceptions of healthcare provision throughout the menopause in the UK: a mixed-methods study. npj Womens Health.

[15] Naslund JA, Marsch LA, McHugo GJ, Bartels SJ (2015). Emerging mHealth and eHealth interventions for serious mental illness: a review of the literature. J Ment Health.

[16] BinDhim NF, Trevena L (2015). There's an app for that: a guide for healthcare practitioners and researchers on smartphone technology. Online J Public Health Inform.

[17] Torous J, Staples P, Shanahan M, Lin C, Peck P, Keshavan M, Onnela J (2015). Utilizing a personal smartphone custom app to assess the Patient Health Questionnaire-9 (PHQ-9) depressive symptoms in patients with major depressive disorder. JMIR Ment Health.

[18] Knowles SE, Toms G, Sanders C, Bee P, Lovell K, Rennick-Egglestone S, Coyle D, Kennedy CM, Littlewood E, Kessler D, Gilbody S, Bower P (2014). Qualitative meta-synthesis of user experience of computerised therapy for depression and anxiety. PLoS One.

[19] Zou P, D'Souza D, Luo Y, Sun W, Zhang H, Yang Y (2022). Potential effects of virtual interventions for menopause management: a systematic review. Menopause.

[20] Rosenstock IM (1974). The Health Belief Model and preventive health behavior. Health Educ Monogr.

[21] Panahi R, Siboni FS, Kheiri M, Ghoozlu KJ, Shafaei M, Dehghankar L (2021). Promoting the adoption of behaviors to prevent osteoporosis using the Health Belief Model integrated with health literacy: quasi-experimental intervention study. BMC Public Health.

[22] Ersin F, Bahar Z (2011). Effect of health belief model and health promotion model on breast cancer early diagnosis behavior: a systematic review. Asian Pac J Cancer Prev.

[23] Jones CJ, Smith H, Llewellyn C (2014). Evaluating the effectiveness of health belief model interventions in improving adherence: a systematic review. Health Psychol Rev.

[24] McGinley AM (2004). Health beliefs and women's use of hormone replacement therapy. Holist Nurs Pract.

[25] Khani Jeihooni A, Mohammadkhah F, Razmjouie F, Harsini PA, Sedghi Jahromi F (2022). Effect of educational intervention based on health belief model on mothers monitoring growth of 6-12 months child with growth disorders. BMC Pediatr.

[26] Walrave M, Waeterloos C, Ponnet K (2020). Adoption of a contact tracing app for containing COVID-19: a health belief model approach. JMIR Public Health Surveill.

[27] Funnell EL, Spadaro B, Martin-Key NA, Benacek J, Bahn S (2024). Perception of apps for mental health assessment with recommendations for future design: United Kingdom semistructured interview study. JMIR Form Res.

[28] El Khoudary SR, Greendale G, Crawford SL, Avis NE, Brooks MM, Thurston RC, Karvonen-Gutierrez C, Waetjen LE, Matthews K (2019). The menopause transition and women's health at midlife: a progress report from the Study of Women's Health Across the Nation (SWAN). Menopause.

[29] StataCorp (2021). Stata Statistical Software: Release 17.

[30] Barrett P (2007). Structural equation modelling: adjudging model fit. Pers Individ Differ.

[31] Schermelleh-Engel K, Moosbrugger H, Müller H (2003). Evaluating the fit of structural equation models: tests of significance and descriptive goodness-of-fit measures. Methods Psychol Res Online.

[32] Vandenberg RJ (2006). Introduction: statistical and methodological myths and urban legends. Org Res Methods.

[33] Joreskog K, Sorbom D (1993). Structural Equation Modelling: Guidelines for Determining Model Fit.

[34] Kline RB (2015). Principles and Practice of Structural Equation Modeling.

[35] Hu L, Bentler PM (1999). Cutoff criteria for fit indexes in covariance structure analysis: conventional criteria versus new alternatives. Struct Equ Model.

[36] Borghouts J, Eikey E, Mark G, De Leon C, Schueller SM, Schneider M, Stadnick N, Zheng K, Mukamel D, Sorkin DH (2021). Barriers to and facilitators of user engagement with digital mental health interventions: systematic review. J Med Internet Res.

[37] Pung A, Fletcher SL, Gunn JM (2018). Mobile app use by primary care patients to manage their depressive symptoms: qualitative study. J Med Internet Res.

[38] Robillard JM, Feng TL, Sporn AB, Lai J, Lo C, Ta M, Nadler R (2019). Availability, readability, and content of privacy policies and terms of agreements of mental health apps. Internet Interv.

[39] Schueller SM, Neary M, Lai J, Epstein DA (2021). Understanding people's use of and perspectives on mood-tracking apps: interview study. JMIR Ment Health.

[40] Torous J, Wisniewski H, Liu G, Keshavan M (2018). Mental health mobile phone app usage, concerns, and benefits among psychiatric outpatients: comparative survey study. JMIR Ment Health.

[41] Spadaro B, Martin-Key NA, Bahn S (2021). Building the digital mental health ecosystem: opportunities and challenges for mobile health innovators. J Med Internet Res.

[42] Hoare E, Milton K, Foster C, Allender S (2016). The associations between sedentary behaviour and mental health among adolescents: a systematic review. Int J Behav Nutr Phys Act.

[43] Rajan T, Menon V (2017). Psychiatric disorders and obesity: a review of association studies. J Postgrad Med.

[44] Alblooshi S, Taylor M, Gill N (2023). Does menopause elevate the risk for developing depression and anxiety? Results from a systematic review. Australas Psychiatry.

[45] Woods NF, Mitchell ES (2016). The Seattle Midlife Women's Health Study: a longitudinal prospective study of women during the menopausal transition and early postmenopause. Womens Midlife Health.

[46] Taylor-Rodgers E, Batterham PJ (2014). Evaluation of an online psychoeducation intervention to promote mental health help seeking attitudes and intentions among young adults: randomised controlled trial. J Affect Disord.

[47] Kravitz RL, Franks P, Feldman MD, Tancredi DJ, Slee CA, Epstein RM, Duberstein PR, Bell RA, Jackson-Triche M, Paterniti DA, Cipri C, Iosif A, Olson S, Kelly-Reif S, Hudnut A, Dvorak S, Turner C, Jerant A (2013). Patient engagement programs for recognition and initial treatment of depression in primary care: a randomized trial. JAMA.

[48] Tang CS, Wong C (2004). Factors influencing the wearing of facemasks to prevent the severe acute respiratory syndrome among adult Chinese in Hong Kong. Prev Med.

[49] Coe AB, Gatewood SBS, Moczygemba LR, Goode JKR, Beckner JO (2012). The use of the health belief model to assess predictors of intent to receive the novel (2009) H1N1 influenza vaccine. Innov Pharm.

[50] Ceasar JN, Claudel SE, Andrews MR, Tamura K, Mitchell V, Brooks AT, Dodge T, El-Toukhy S, Farmer N, Middleton K, Sabado-Liwag M, Troncoso M, Wallen GR, Powell-Wiley TM (2019). Community engagement in the development of an mHealth-enabled physical activity and cardiovascular health intervention (Step It Up): pilot focus group study. JMIR Form Res.

[51] Fortuna KL, Naslund JA, LaCroix JM, Bianco CL, Brooks JM, Zisman-Ilani Y, Muralidharan A, Deegan P (2020). Digital peer support mental health interventions for people with a lived experience of a serious mental illness: systematic review. JMIR Ment Health.

