# Supplementary material for: Intention to Use a Mental Health App for Menopause: Health Belief Model Approach
Source: JMIR Form Res. 2024 Oct 16;8:e60434. doi: 10.2196/60434 (PMC11525080; doi:10.2196/60434)
Supplement: Multimedia Appendix 1 [file formative_v8i1e60434_app1.docx]

**Supplementary Materials.**

**
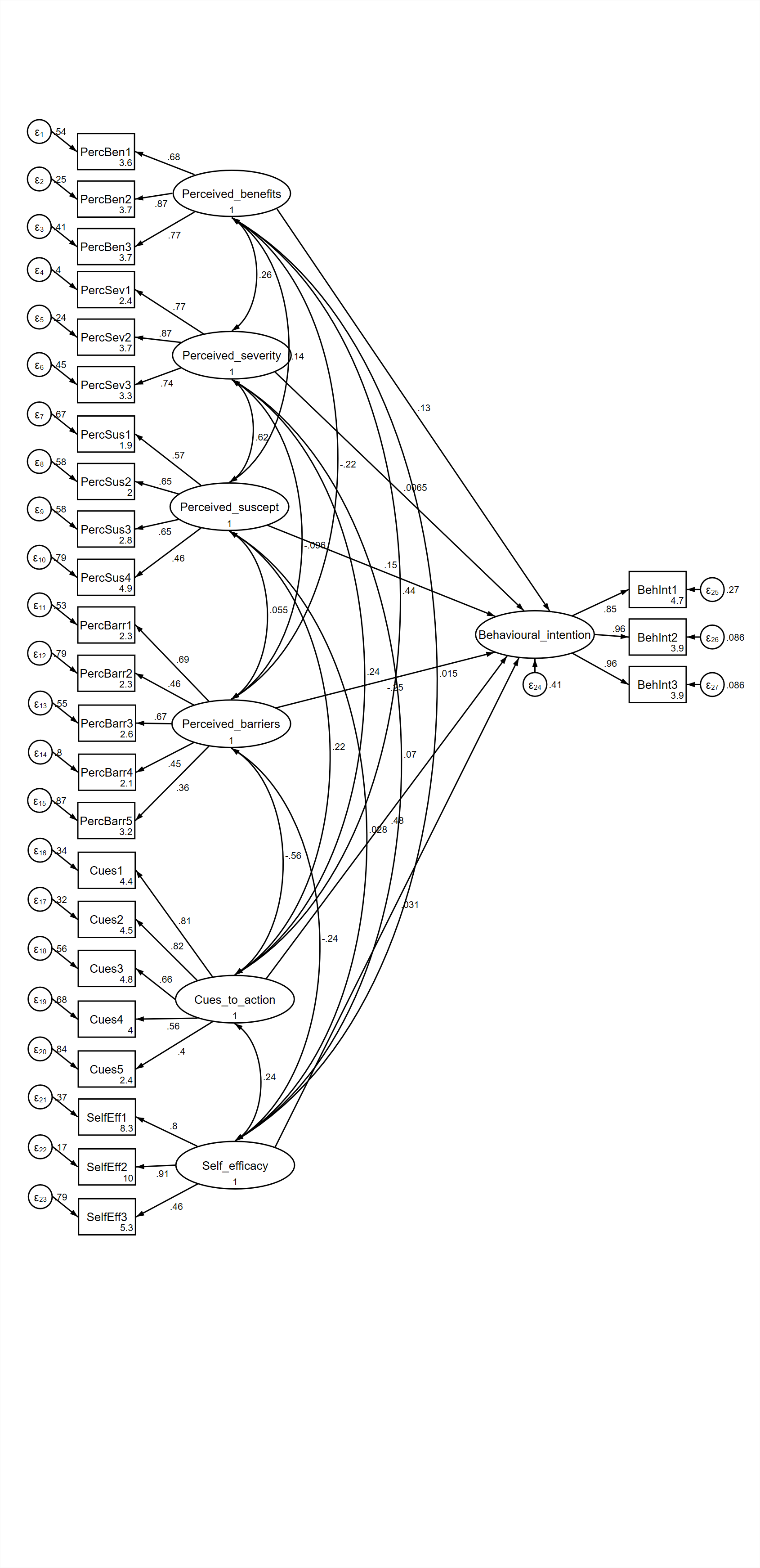
**

**Figure S1.** Model schema of HBM constructs predicting intention to use an app for mental health (including all covariates)
